# Toward Patient-Centric Digital Monitoring of Obstructive Sleep Apnea: Mixed Methods Study

**DOI:** 10.2196/82460

**Published:** 2026-01-08

**Authors:** James Kenneth Timmis, Kerstin Alexandra Schorr, Rana Yüksel, Tim van den Broek, Sebastiaan Overeem, Dagmar Josine Smid, Willem Johan van den Brink, Nina Leonie Haring

**Affiliations:** 1 Department of Political Science University of Freiburg Freiburg, Baden-Wurttemberg Germany; 2 Athena Institute for Research on Innovation and Communication in Health and Life Sciences Vrije Universiteit Amsterdam Amsterdam, North Holland The Netherlands; 3 Research Group Microbiology and Systems Biology Netherlands Organization for Applied Scientific Research (TNO) Leiden The Netherlands; 4 Sleep Medicine Center Kempenhaeghe Heeze The Netherlands; 5 Department of Electrical Engineering Eindhoven University of Technology Eindhoven The Netherlands; 6 Research Group Human Performance Netherlands Organisation for Applied Scientific Research (TNO) Soesterberg The Netherlands

**Keywords:** obstructive sleep apnea, digital biomarkers, remote patient monitoring, patient-centric care, wearable sensors, digital outcome measures, patient-reported outcomes

## Abstract

**Background:**

Obstructive sleep apnea (OSA) is a sleep disorder characterized by repeated breathing disruptions during sleep. Remote patient monitoring (RPM) of OSA is important, yet contemporary methods are limited. Sensor-based digital health technologies (sDHTs) promise a step advance in OSA RPM, but must provide meaningful, actionable, and usable outputs for patients. While the centrality of considering patient views in sDHT development is widely acknowledged, patient perspectives and priorities are rarely assessed.

**Objective:**

This study aimed to identify patient-prioritized health aspects and preferences for digital measures and RPM to enhance OSA care quality and patient experience, guided by the digital measures that matter framework.

**Methods:**

We used a mixed methods design combining quantitative and qualitative approaches. Individuals with a formal OSA diagnosis and persistent sleep problems (n=223) completed a survey in which they ranked items related to treatment burdens and health priorities, and responded to open-ended questions about restoring previous quality-of-life elements and desired health goals. To gain deeper qualitative insights, we conducted semistructured interviews with patients with OSA, patient advocates, and health care professionals (n=11), focusing on follow-up care, attitudes toward sDHTs and RPM, and preferences for future OSA-related sDHTs and metrics. Quantitative data were analyzed using bootstrap-aggregated Borda counts (broad support) and Plackett-Luce modeling (intense prioritization), while qualitative data from surveys and interviews were analyzed thematically.

**Results:**

Key meaningful aspects of health included the improvement of subjective sleep quality (top-ranked burden; health goal for 46.5%, 93/200 of participants), an increase in daytime energy (quality-of-life aspect to restore for 35.6%, 72/202 and health goal for 25.5%, 51/200 of participants), and physical activity (quality-of-life aspect to restore for 24.7%, 50/202 and health goal for 16.5%, 33/200 of participants). Sleep characteristics and daytime energy were priority targets for digital measure development. Smartwatches, sleep mats, and smart rings were preferred modalities for integration into RPM. Participants’ priorities for enhancing monitoring included (1) expanding metrics beyond the Apnea-Hypopnea Index (AHI; 36.6%, 52/142), (2) improving measurement accuracy (20.4%, 29/142), and (3) ensuring outputs are meaningful, understandable (18.3%, 26/142), and actionable (9.2%, 13/142). Patients also reported difficulty interpreting RPM data to determine if and when follow-up care is needed and what type of care is appropriate.

**Conclusions:**

RPM solutions for OSA should expand beyond AHI, ensure accuracy and interpretability, and provide actionable insights to support comprehensive patient-centric management.

## Introduction

Obstructive sleep apnea (OSA) is a sleep-related breathing disorder characterized by repeated episodes of partial or complete blockage of the upper airway during sleep, leading to oxygen desaturation events and reduced sleep quality [1]. Common symptoms include loud snoring, waking up gasping for air or choking, and excessive daytime sleepiness. OSA is a chronic condition, and approximately 14% of the global population is affected [2]. Due to aging populations and increasing rates of obesity, the prevalence of OSA is expected to rise in the upcoming years. The chronic nature of OSA, combined with its links to other health conditions and multimorbidity, imposes a high burden on patients and significantly strains health system resources [3,4].

The Apnea-Hypopnea Index (AHI) is the standard for classifying OSA severity by counting the number of apneas (complete pauses in breathing) and hypopneas (partial reductions in breathing) per hour of sleep. While AHI is widely used, it has limitations, such as not accounting for the duration and depth of breathing interruptions, or their impact on oxygen levels and sleep stages [5]. Consequently, AHI scores show poor correlation with disease burden and treatment outcomes [6,7]. Moreover, AHI lacks meaning from the patient perspective, not only because it fails to capture symptoms or daily functional impairments, but also because many patients struggle to interpret clinical measures and understand what pertinent changes in their scores actually imply for their health [8].

The most common treatment options for OSA (in the Netherlands) include continuous positive airway pressure (CPAP) and mandibular advancement devices (MADs) [9]. CPAP is routinely supported by remote patient monitoring (RPM) solutions. RPM refers to systems that allow health care professionals to assess, monitor, and care for patients virtually, often in extraclinical settings [10]. While CPAP RPM platforms provide data on use, mask leaks, and AHI scores [11], they fail to capture patient-centric outcomes, and often do not prevent nonadherence [12]. In contrast, most MADs lack embedded sensors, so adherence and treatment effectiveness are typically assessed subjectively during follow-up visits [13].

Patient-reported outcome measures (PROMs) offer invaluable insights into the subjective experiences of individuals with OSA, especially regarding disease-related quality of life [14]. However, PROMs are limited by respondent burden and various biases, such as nonresponse, fatigue, and recall bias [15,16]. Sensor-based digital health technologies (sDHTs) can be defined as (often wearable) devices that use sensors (for instance, accelerometers or photoplethysmography) to capture health measures, such as symptoms and functional states, continuously [17,18]. They have the potential to (partially) replace, or complement, existing PROMs and thereby provide more objective, real-time insights into patients’ health [19,20]. In the context of OSA, sDHTs may be integrated directly into treatment devices or used independently (eg, watches and sleep mats). By passively and continuously capturing objective, longitudinal data based on digital biomarkers, sDHTs also hold potential to facilitate the prediction of treatment responses, optimization of titration, and enhancement of adherence. This supports the transition toward person-centered OSA care, which empowers patients to manage their own condition [21]. Additionally, patient-centric digital end points are becoming increasingly important for clinical research [22].

However, studies across a wide range of different settings have shown that, for (new generations of) technologies to be properly adopted, patients must consider them meaningful, actionable, and usable [18,22-25]. Although the importance of considering patient priorities in sDHT development is widely acknowledged, the former are rarely assessed and integrated in new sDHTs [26]. Efforts led by the Digital Medicine Society (DiMe) are paving the way for the development of sDHTs that are truly patient-centric [18,23]. According to DiMe’s digital measures that matter framework, this process begins with the identification of meaningful aspects of health—aspects of a condition that patients wish to improve, arrest, or prevent—which then guide the selection of measurable concepts that reflect patients’ lived experiences and priorities [18]. Noteworthy is that the perspectives of patients with OSA are rarely reported in scientific literature, and we are not aware of literature published on the priorities of patients with OSA for sDHTs. To contribute toward addressing this knowledge gap, this study aimed to identify meaningful aspects of health, patient and clinician priorities, and preferences regarding sDHTs for OSA management using a mixed methods design.

## Methods

### Study Design

This observational, exploratory study used a sequential mixed methods design, integrating survey-based quantitative data with qualitative data collected in semistructured interviews to gather insights from individuals with OSA reporting persistent sleep problems and health care professionals involved in OSA care. Quantitative data were collected through a survey completed by n=223 respondents (which is a sample size considered sufficient in published studies with similar study aims, designs, and settings) [27-29], between January and March 2024, while qualitative insights were obtained from semistructured interviews conducted in May-June 2024 with 6 patients, 2 patient advocates, and 3 health care professionals (Table S1 in Multimedia Appendix 1).

### Survey

The survey targeted a broad population of individuals with self-reported sleep problems; participants were included who were aged 18 years and older and experiencing sleep problems at least 3 times per week for a minimum of 3 consecutive months. Moreover, we included in this analysis only those respondents who reported receiving a formal OSA diagnosis from a health care professional. Survey participants were recruited via social media posts, flyers distributed in primary and secondary sleep care settings (including physiotherapy practices and sleep clinics), and a newsletter announcement by the Dutch Apnea Association (ApneuVereniging). The survey (originally developed in Dutch and translated into English for this manuscript) consisted of 18 questions (multiple choice, ranking, and open-ended) distributed through the online platform Survalyzer (Multimedia Appendix 1). Respondents were only able to participate after providing informed consent. The survey included 3 themes. The first theme covered demographic and background information, including medical history and sleep disorder profile (questions 1-8). Questions 1 to 4 were adapted from the “Netherlands working conditions” survey conducted by the Netherlands Organization for Applied Scientific Research (TNO) and Statistics Netherlands [30]. Questions 5 to 8 were developed based on relevant literature and expert input from sleep health care professionals. The second theme focused on meaningful aspects of health (questions 9-12), with questions developed using the digital measures that matter framework by Manta et al [18], which provides patient-centered question formulations to identify aspects of health most meaningful to individuals. The third theme explored preferences and experiences with sDHTs, adapted to the sleep field from a survey exploring this theme in patients in the cardiovascular risk management care pathway, using expert input and supporting literature. The original survey is currently being prepared for publication. In open-ended questions, participants were asked to provide details on, for example, health goals or the aspects that positively drove their quality of life (before their development of OSA), which they would like to restore. Participants had no word limit. While pretesting is a standard step to ensure clarity and reliability, it was not feasible in this instance due to time constraints. Face validity of the survey was assessed by an internal panel of experts experienced in survey design.

### Survey Data Analysis

The data analysis for this study primarily involved descriptive statistics, including the calculation of frequencies and percentages to summarize the data and identify patterns and trends within the dataset. Ranking items were analyzed using both Borda counts and the Plackett-Luce model. Using both methods allowed us to combine the accessibility of Borda counts with the statistical rigor of Plackett-Luce and to assess consistency across approaches. The Borda count is a point-based voting method in which each item receives a score based on its rank position, with scores aggregated across participants to produce a consensus ranking [31]. We included Borda counts because they provide a simple and easily interpretable descriptive summary of rankings that has been widely used in previous work. Within each question, we calculated mean scores and 95% CIs. This method assumes complete rankings from all participants. However, some ranking questions in our survey elicited partial rankings, as participants were asked to rank only their top 3 items from a larger set, potentially introducing bias. To address this, the Plackett-Luce model was used as a complementary approach, as it does not penalize unranked items [32]. The Plackett-Luce model estimates the relative *worth* or preference strength of each item based on observed rankings, including partial ones. Each item is assigned a positive worth parameter, which is interpreted comparatively: an item with a higher worth than others is more likely to be systematically preferred. Observations were treated as independent despite potential within-subject correlation, as sparse data precluded models accounting for this, and results should be interpreted accordingly. All computations were performed in R (version 4.4.0; R Foundation for Statistical Computing) using the *PlackettLuce* (version 0.4.3) and *emmeans* (version *1.11.1*) packages.

Open-ended survey responses were analyzed using thematic analysis as outlined by Braun and Clarke [33]. Coding was performed by one researcher and independently reviewed by a second researcher to ensure consistency. Themes were developed based on the frequency, emphasis, and contextual richness of participant responses. In some cases, subdividing themes into subthemes was necessary to provide a deeper understanding of the data.

### Interviews

We used purposive sampling to select adequate participants for the interviews. The inclusion criteria were as follows: Dutch patients who have been formally diagnosed with OSA and have experience with (digital) RPM for OSA (current or discontinued use; the latter, to elicit challenges for general use and adherence); health care professionals with a specialization in OSA and who have experience with (digital) RPM for OSA, to elicit their perspective on clinical workflows and patient interactions; and patient advocates for OSA, to elicit perspectives on the pain points and needs of the broader community of patients with OSA in the Netherlands. Our sample included individuals from (1) patients with OSA from the survey who provided email addresses for follow-up, (2) TNO’s network from previous OSA collaborations, and (3) clinicians identified through online searches for OSA expertise. The recruitment email included detailed information on the study, including the background of the research, its objectives, and the informed consent form. If individuals agreed to participate, they were able to choose between an in-person or online interview via Microsoft Teams.

The interviews, which on average took about 60 minutes, were conducted and recorded via Microsoft Teams by RY, who used an interview guide that had been expert checked (by NLH and JKT) and piloted a priori (Multimedia Appendix 1). The interview guide was based on a conceptual framework closely aligned with the technology acceptance model, which had been adapted to systematically elicit the perceived usefulness and ease of use of, perceived needs for, and willingness to engage with OSA RPM and pertinent communication and reporting mechanisms in inter alia OSA follow-up care. The conceptual framework was also used to create the initial deductive code book.

### Qualitative Data Analysis

The interviews were manually transcribed (verbatim) by RY. To support analysis, the transcripts were imported into the computer-assisted qualitative data analysis software (CAQDAS) Atlas.TI (version 8.02; Scientific Software Development). The transcripts were analyzed by RY based on the 6 steps of thematic analysis using deductive and inductive (complementary) coding approaches as outlined by Braun and Clarke [33]. To improve the credibility of the analysis, 10% of the transcripts (in this case, n=2 interviews) were independently coded by DS, see acknowledgments. We determined intercoder reliability (approximately 90%) by manually reviewing the codes created and assigned by both coders for both interview transcripts. To increase dependability, codes were carefully tabulated and aggregated into themes based closely on the initial conceptual framework, analyzed and discussed with NLH and JKT, and finally reported by RY. RY also performed member checks (providing a summary of the preliminary analysis of an interview together with a couple of key quotes to the pertinent interviewer, and asking for comment) with multiple interviewees to improve confirmability. The code book and coding were reviewed by another researcher. Finally, the process of selective coding focused on selecting the most important and representative codes to develop overarching themes that addressed the research subquestions of this study [34]. Atlas.Ti was used for the coding process. While the number of possible interviews was limited by time and resource constraints, we, by tendency, reached data saturation in interview 7. However, one further major concept was revealed in interview 9. Interviews 10 and 11 revealed no additional concepts. In consequence, data saturation can therefore not be considered formally achieved. To enhance rigor, we used member checks, peer debriefing, and strategies to minimize social desirability and interview bias (eg, building rapport). For the qualitative component of this study, we carefully considered the 4 established quality criteria of trustworthiness in qualitative research, namely credibility, transferability, dependability, and confirmability, to enhance the overall quality of the study [35].

### Ethical Considerations

This study was reviewed in accordance with institutional and national ethical standards for research involving human participants. The research protocol was submitted to TNO’s ethical review board, and ethical approval was obtained from TNO’s ethical review board for both the survey (study 2023-103) and the interviews (study 2024-026). Informed consent was obtained from participants before data collection. Participants received no compensation for participation. Data used in this study were anonymized, and no personally identifiable information was retained. All data were stored on secure, access-controlled servers in compliance with data protection regulations. No identifiable images or personal information of participants are included in this manuscript or supplementary materials. Ethical approval was further granted by TNO’s ethical review board for making data available in a repository (study 2023-103).

### Data Management and Availability

The datasets generated and analyzed during this study are publicly available in the Harvard Dataverse repository under the title “Replication Data for: Toward Patient-Centric Digital Health Solutions for Obstructive Sleep Apnea Monitoring: Perspectives from Dutch Patients and Healthcare Professionals – a mixed-method study” [36]. The repository includes an anonymized survey dataset (n=223) containing quantitative responses on meaningful aspects of health, attitudes toward remote patient monitoring, and digital health technology preferences. To protect participant confidentiality, direct identifiers have been removed, and free-text fields have been redacted to avoid inadvertent disclosure of personal information.

### Code Availability

The analysis code used to generate the quantitative results reported in this study is publicly available in the same Harvard Dataverse repository [36].

### Reporting Guideline

This study adhered to the Mixed Methods Reporting in Rehabilitation and Health Sciences guideline, and the completed checklist is provided in Multimedia Appendix 2.

## Results

The survey focused on the themes of meaningful aspects of health, current use of and attitudes toward sDHTs or RPM, and preferences for future OSA-specific sDHTs and RPM solutions. To gain deeper qualitative insights, we also conducted semistructured interviews with formally diagnosed patients with OSA, patient advocates, and health care professionals. The interviews covered the themes follow-up care, attitudes toward sDHTs and RPM, and preferences for future OSA-related sDHTs and metrics; Table S2 in Multimedia Appendix 1.

### Demographic Information

A total of 404 individuals initiated the survey; after applying eligibility criteria, the final analytic sample comprised 223 Dutch patients with a formal OSA diagnosis (Multimedia Appendix 3). A total of 48.4% were female, and the mean age was 65 (SD 9) years (Table 1). The majority of the sample (133/223, 59.6%) was highly educated, and 67.3% (150/223) of the participants worked for less than 1 day per week or not at all. A total of 34.1% (76/223) were formally diagnosed with at least one other sleep-related illness besides OSA. Also, other comorbid conditions were reported by 71% (158/223), with obesity and cardiovascular disease being the most prevalent. All interview participants were Dutch and purposefully selected; they had to be at least 18 years of age and have preexisting experience with OSA RPM solutions (Table S1 in Multimedia Appendix 1). We interviewed 6 Dutch individuals with a formal OSA diagnosis, 2 patient advocates affiliated with the national association for patients with OSA (ApneuVereniging), and 3 health care professionals experienced with managing OSA. This purposive sample was designed to capture a range of perspectives from key stakeholder groups (patients, advocates, and clinicians; Table S2 in Multimedia Appendix 1).

**Table 1:** Respondent characteristics of surveyed cohort (n=223).

Respondent characteristics		Values
Age (years), median (range)		67 (28-86)
**Sex, n (%)**		
	Male	48 (108)
	Female	52 (115)
**Education, n (%)**		
	Secondary education	6.7 (15)
	Secondary vocational education	33.6 (75)
	Higher professional education and academic education	59.6 (133)
**OSA care provider visited within the last year, n (%)**		
	General practitioner	38 (84)
	Medical specialist (outside sleep clinic)	50 (111)
	Company doctor	6 (13)
	Psychologist	6 (13)
	Sleep therapist	4 (9)
	Sleep clinic	27 (59)
	Other	3 (6)
	None	30 (67)
**Sleep-related diagnoses, n (%)**		
	Obstructive sleep apnea	100 (223)
	Insomnia	10 (22)
	Hypersomnia	12 (27)
	Sleep rhythm disorder	1 (2)
	Parasomnia	4 (10)
	Sleep-related movement disorder	15 (34)
	Other	3 (6)
**Other diagnoses, n (%)**		
	Obesity	33 (73)
	Cardiovascular disease	37 (82)
	Diabetes type 2	15 (34)
	Depression	9 (20)
	Other	18 (42)
	None	29 (64)

### Meaningful Aspects of Health

Overall, we found that meaningful aspects of health for individuals with OSA and persistent sleep problems encompass both physical limitations and psychological burdens. Subjective sleep quality, daytime energy levels, and physical activity consistently emerged as key priorities—highlighted as important, considerable burdens when impaired, and as goals for improvement or resumption. In addition, psychological concerns such as worrying about health impacts and difficulties concentrating reflect the broader mental burden of OSA. We present results from ranking exercises of prespecified health aspects and, separately, thematic analyses of responses to open-ended questions.

### Burdens of Living With OSA (Ranking)

**Figure 1:**
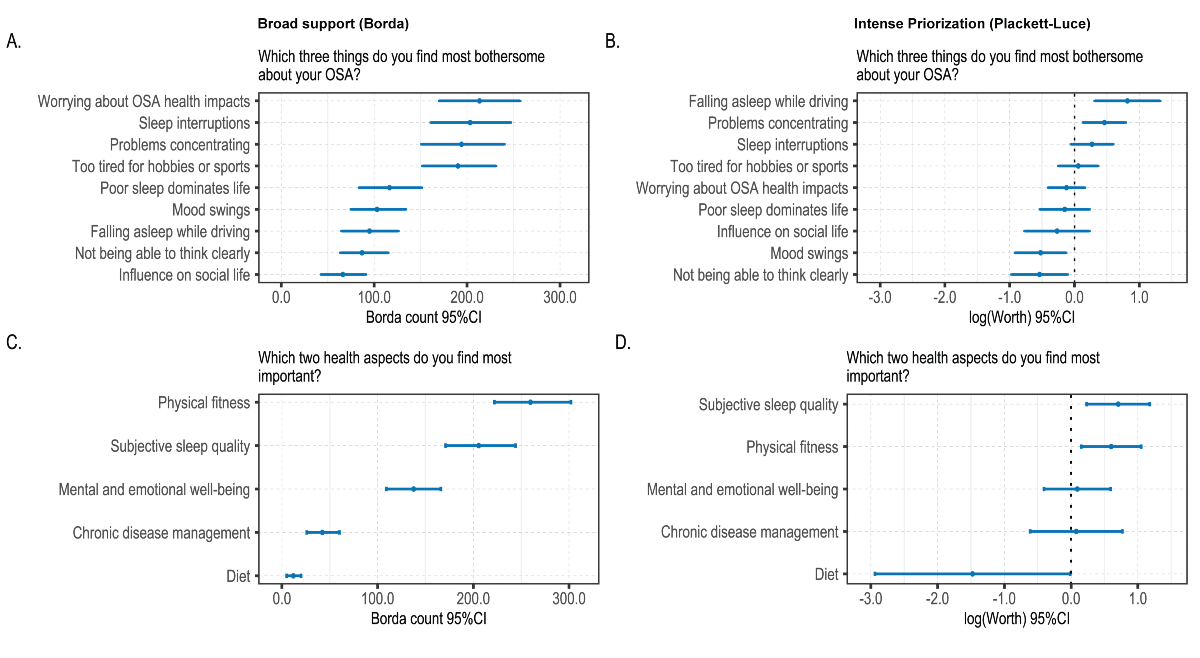
Meaningful aspects of health identified through survey questions. Parts A and B were derived from a 3-item ranking question from 9 prespecified items (n=221). Parts B and C were derived from a 2-item ranking question from 5 prespecified items (n=219). OSA: obstructive sleep apnea.

Worrying about OSA health impacts, sleep interruptions, and problems concentrating were ranked highest (Figure 1, parts A and B). Based on Borda counts, these items showed broad support across the cohort, while Plackett-Luce modeling revealed that certain concerns—such as falling asleep while driving or problems concentrating – were intensely prioritized by subsets of respondents. Being too tired for hobbies or sport was also considered an important burden (fourth place for both methods).

### General Health Priorities (Ranking)

While physical fitness attracted the strongest support overall, it came in second regarding rank concentration (Figure 1, parts C and D). This means that while physical fitness was considered most important overall, the agreement on specific ranks selected was less consistent than, for example, mental and emotional well-being or chronic disease management. Subjective sleep quality was ranked second overall, and first, based on rank agreement (Figure 1, part D), so the reversed relationship was observed when compared with physical fitness. Subjective sleep quality refers to the experienced quality of sleep as perceived by the individual, including aspects such as sleep fragmentation, feelings of restorative sleep, and, more generally, what respondents described as “good” sleep without providing a fixed definition. Mental and emotional well-being, chronic disease management, and diet came in third, fourth, and fifth, respectively, for both analytical methods, respectively.

### Restoring Previous Quality of Life (Open-Ended)

A broad desire for higher daytime energy levels was expressed by 35.5% (72/202) of respondents (Table 2). For example, one participant reported that they missed “getting through a whole day without constraints from extreme tiredness.” Another explained: “I still do all things normally, only it [takes much longer] because you are so terribly tired.” Various types of physical activities were described by 24.8% (50/202) of respondents as aspects that they missed from their quality of life before disease onset, such as hiking, cycling, or exercise more generally. Interestingly, “None” was the third most common theme (17.8%, 36/202), reflecting respondents who reported nothing from their previous quality of life they wished to restore. Social activities (16.8%, 34/202), activities requiring concentration, such as reading a book (15.8%, 32/202), and restorative sleep (14.9%, 30/202) were also mentioned.

**Table 2:** Aspects of their previous quality of life that respondents wished to restore (derived from open-ended questions; n=202).

Items	Respondents, n (%)
Having more energy	72 (35.6)
Physical activity	50 (24.8)
None	36 (17.8)
Social life and leisure	34 (16.8)
Concentration	32 (15.8)
Subjective sleep quality	30 (14.9)
Feeling more productive	22 (10.9)
Daily activities	16 (7.9)
Physical health	16 (7.9)
Being intervention-free	3 (1.5)

### Health Goals Relating to OSA Symptoms (Open-Ended)

As shown in Table 3, “Subjective sleep quality” was the top health goal for our sample (46.5%, 93/200). For example, one participant explained that their goal was: “no more lying awake after going to the toilet at night.” The themes weight loss and daytime energy levels both came in second (25.2%, 51/200). Participants explained their desire to feel less tired and more energetic throughout the day. The third most frequently mentioned aspect was physical activity (16.3%, 33/200). Additionally, 14.4% (29/200) of participants wish to improve “health aspects related to physical health,” including, for example, addressing night sweats, palpitations, or restless leg syndrome.

**Table 3:** Health goals related to obstructive sleep apnea symptoms derived from open-ended questions (n=200).

Items	Respondents, n (%)
Subjective sleep quality	93 (46.5)
Daytime energy levels	51 (25.2)
Weight loss	51 (25.2)
Physical activity	33 (16.3)
Physical symptoms	29 (14.4)
Improved treatment	22 (10.9)
Concentration	16 (7.9)
Mental health	13 (6.4)
Social life and leisure	8 (4)
Other	5 (2.4)
None	3 (1.5)

### OSA Follow-Up Care in the Netherlands

Based on the survey data, half of the participants reported contact with medical specialists, and 38% with general practitioners, respectively. A total of 30% (67/223) indicated that they had no physical follow-up health care visits. 26.4% (59/223) indicated that they had visited sleep centers during the past year (Table 1). Notably, several respondents who selected the option “other” explained that they had not attended physical follow-up visits but were, instead, relying on remote monitoring by their CPAP devices or self-monitoring of their condition. For example, one participant explained:

No. I haven’t seen a specialist since I got the [CPAP device]. According to the pulmonologist, everything was under control, and there was no need to return.

According to some interviewees, follow-up care is straightforward but limited, as exemplified here by the following comment:

[I was] diagnosed [with] sleep apnea [and] provided...with the CPAP mask. A year later, I had a follow-up appointment...to ensure my mask fit well and that everything was in order. After that, I received no follow-up care anymore.

Participant B8; patient with OSA who is also a general practitioner

B3 (patient with OSA) highlighted that they were not even sure what type of care they should be receiving: “I received no follow-up care. I don’t even know what follow-up care you should get.” Participant B11 (a patient advocate) explained that this was likely due to resource constraints:

Clinics don’t have the capacity to provide follow-up care for all these patients. There are long waiting lists, and those waiting for OSA diagnosis are prioritized, meaning that those who should receive follow-up care need to wait.

Participant B11; patient advocate

By the same token, the two interviewed somnologists highlighted that, on the one hand, patients have a role in taking charge if issues occur, and, on the other, follow-up care can indeed vary greatly based on patients’ needs and the severity of their OSA. Participant B7 (a somnologist) explained, “Doctors expect that patients will initiate contact if there’s a problem....Effective communication and reporting in follow-up care...also requires patients to allocate time.” Participant B9 (pulmonologist/somnologist) provided a top-level view of her patient-centered approach to follow-up care:

I do an initial evaluation after 6-8 weeks post-diagnosis, then plan further check-ups based on the patient’s condition and needs. If a patient is doing well, I schedule a follow-up after a year..... If there are any issues, I see them sooner, using RPM data from their CPAP device to guide decisions.

Participant B9

Nevertheless, participant B5 (a patient with OSA) explained, other hurdles might exist in achieving good continuity of care: “I called my supplier, who said that they had sent a report of my monitored data to the hospital, but the hospital claimed they that hadn’t received any report from my supplier.”

### sDHTs Used and Attitudes Toward RPM

**Figure 2:**
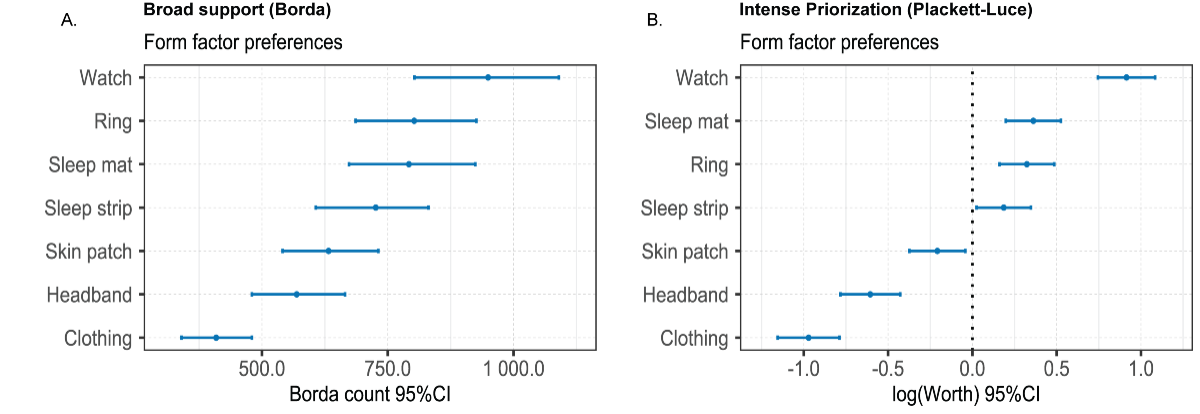
Form factor preferences. Parts A and B were derived from the full ranking of 7 prespecified items (n=109).

Based on the survey data, 86% (182/212) of participants agreed that sDHTs could contribute toward improving management of their OSA. Table 4 shows that, in our sample, the most frequently used device for self-monitoring was CPAP (87.1%, 155/178), followed by weight scales (47.8%, 85/178), blood pressure monitors (46.6%, 83/178), and smart watches (35.4%, 63/178 for heart rate measurements and 19.7%, 35/178 for sleep tracking). Sleeping mats were used least frequently (n=1). Figure 2, parts A and B, shows that, when asked to rank different form factors according to their preference, smart watches were ranked first, both in terms of overall frequency and subgroup concentration. The least preferred technology was clothing with integrated sensors. Notably, only a single participant reported using a sleep mat, yet when participants were asked to rank their preferred technologies, sleeping mats were ranked among the top three. While the majority of participants (67%, 119/178) agreed that both they themselves, as well as their care providers, should have access to data collected with sDHTs, 28% (49/178) thought their care providers should have access only under certain conditions—merely 4% (7/178) indicated that they should be the only party with access to their data.

Also, the majority of our interviewees held positive views toward the usability of RPM technologies for supporting OSA management. Participant B6 (a patient with OSA), for example, was particularly enthusiastic: “RPM tools [are] a great addition because of the shortage of health care professionals now, so I think it’s fantastic that we’re focusing on that.” By the same token, some patients with OSA had concerns regarding the dependability of the RPM ecosystem and the actionability of information contained therein. For example, a patient with OSA stated:

I appreciate RPM, but it’s only effective if I receive feedback from the RPM technology supplier or hospital. Ideally, it should alert you promptly if issues like stopped breathing arise, which can ensure constant surveillance and timely intervention.

Participant B3; patient with OSA

Moreover, there exists an unmet need regarding the interpretation of data: A patient with OSA explained:

I do see my sleep apnea values, for example, an AHI score of 11.3, [but] is that good? What does this score mean for my health, and how can I improve it? I’m missing this information, and I think many do.

Participant B4; patient with OSA

**Table 4:** Currently used technologies to monitor health are derived from multiple-choice questions (n=178).

Items	Respondents, n (%)
CPAP^a^	155 (87.1)
Weight scale	85 (47.8)
Blood pressure monitor	83 (46.6)
Smartwatch (heart rate)	63 (35.4)
Pulse oximeter	46 (25.8)
Smartwatch (sleep)	35 (19.7)
Health app	21 (11.8)
Glucose monitor	16 (9)
Other	5 (2.8)
Sleep mat	1 (0.6)

^a^CPAP: continuous positive airway pressure. Images of devices are shown in Multimedia Appendix 1.

This was somewhat echoed, albeit in more general terms, by the somnologist (Participant B7), who was of the view that current RPM solutions have not yet reached the degree of maturity required for effective OSA remote management:

Follow-up care cannot be provided as effectively through digital health technologies like RPM compared to clinical care. As a doctor, you need to see, touch, and smell a patient to provide optimal care. You miss the human aspect of care when everything is digitized and managed through technologies that provide RPM.

Participant B7

Nevertheless, our data indicate that some patients are actively engaged with their RPM data. Participant B2 (a patient with OSA) explained:

I read my reported data from the screen of my device. Then I have the DreamMapper app [patient-facing app showing CPAP data]. I take out the SD card of my [CPAP] device and plug it into my computer, on which I have installed the Oscar program. This program gives me a comprehensive report on my monitored sleep apnea. Oscar is usually a very good program, but the hospitals don’t want to use it because it’s not validated.

Participant B2

This quote quite vividly illustrates that, while solutions exist that can be meaningful for patients with OSA who want to dive deeper into their data and better understand their disease, these might be located outside of traditional clinical care pathways (and therefore lack clinical oversight and supervision).

### Improving Digital Measures for the Future of OSA RPM

**Figure 3:**
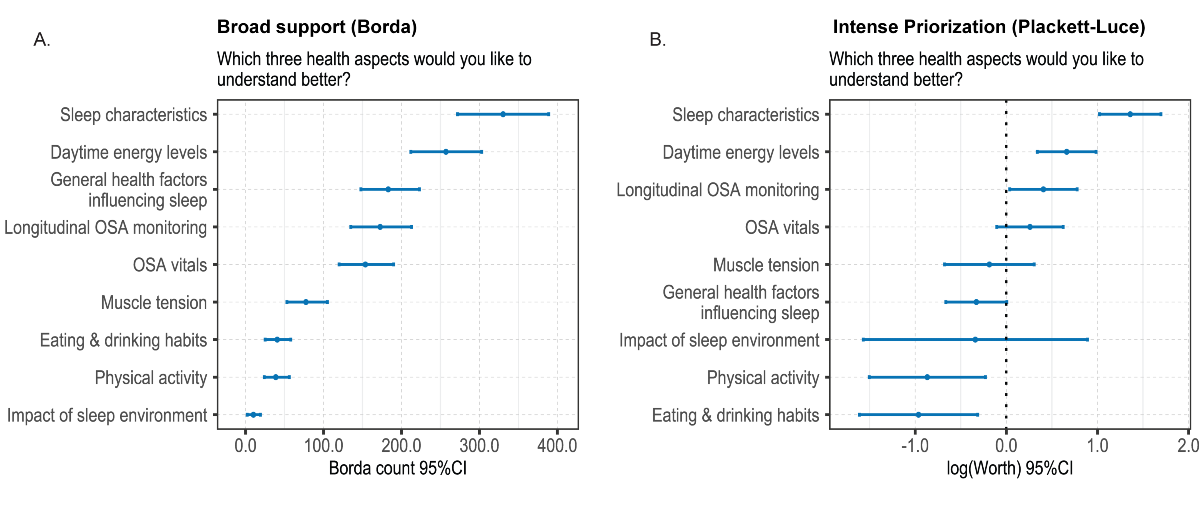
Health aspects participants would like to understand better. Parts A and B were derived from a 3-item ranking of 9 prespecified items (n=213). OSA: obstructive sleep apnea.

Participants were asked to rank which three digital measures would help them gain more insight into their OSA. Sleep characteristics ranked highest overall. However, broad support in the Borda count analysis and rank agreement in the Plackett-Luce model for both sleep characteristics and daytime energy levels (second rank) indicate that these are the top targets for future digital measures (Figure 3, parts A and B). Physical activity was the only other item that came in at the identical rank (8) for both methods of analysis. When asked how current OSA measurement practices could be improved, the most frequently given answer related to suggestions for additional measurements (mentioned by 52/142, 36.6% of respondents; Table 5). Specifically, the majority of survey participants were interested in OSA vitals beyond AHI, such as heart rate–derived and saturation-based measures. Obtaining insights into sleep characteristics, such as sleep stages, was also mentioned by several survey participants. Other suggestions included improved accuracy and reliability of measurements, and a more comprehensible presentation of monitoring data. For example, one survey participant highlighted the need for more clarity in analysis reports, such as presentation in layman’s terms, whereas another patient expressed the desire for more monitoring and guidance from OSA professionals and home care providers. Also, the more general value of digital measures for OSA monitoring was underscored. For example, one survey participant indicated that they would like to add monitoring options to their MAD-based treatment:

Many people have MADs but have no proof – except through complaints (snoring) or feeling fit – [indicating] whether it helps..... It would be great if...[a wearable or smart watch], or other technology could do that

track and report

**Table 5:** Suggestions for improving obstructive sleep apnea remote patient monitoring. Thematically analyzed responses to an open-ended question. Obstructive sleep apnea management advice includes lifestyle advice and contact with a health care provider (n=142).

Items			Respondents, n (%)
**Additional measurements**			
	All	52 (36.6)	
	OSA^a^ severity measures^b^	21 (40.4)	
	Sleep characteristics	16 (30.8)	
	Metabolic health	11 (21.2)	
	Unspecified	4 (7.7)	
None			32 (22.5)
Accuracy and reliability			29 (20.4)
More comprehensible presentation			26 (18.3)
OSA management advice			13 (9.2)
Product design			5 (3.5)
Other			6 (4.2)

^a^OSA: obstructive sleep apnea.

^b^OSA severity measures: heart rate–derived measures, breathing, saturation.

The interview data provided additional detail on a range of potential improvements for future OSA management. The majority of patients with OSA preferred noninvasive sDHTs and emphasized the need for a stronger product design focus on less disruptive and more convenient devices for daily use, and better integration with daily attire. A patient with OSA explained:

I already use a [smart watch]...for my heart monitoring, but it would be nice if future RPM technologies are non-invasive, like an App or a small device beside the bed. Large or intrusive devices are uncomfortable for light sleepers like me.

Participant B6; patient with OSA

Participant B4 (a patient with OSA) also mentioned the, inter alia, visual product design of wearable RPM technology: “A smartwatch-like device that tracks my heart rate and oxygen saturation would be ideal, as long as it’s non-invasive and looks good.” Regarding specific measures, participant B4 (a patient with OSA) said*:* “I would like to measure my oxygen saturation during the night. This could be an improvement regarding RPM metrics for OSA.”

## Discussion

### Principal Findings

This mixed methods study, guided by DiMe’s digital measures that matter framework, aimed to, first, identify meaningful aspects of health and care needs of individuals with OSA, and, second, inform the improvement of existing, and the development of new, pertinent digital measures and RPM solutions. Based on our two Dutch cohorts (patients, their representatives, and specialized health care professionals), our principal findings are that, first, improving subjective sleep quality, increasing physical activity, and increasing daytime energy levels are key meaningful aspects. Second, sleep characteristics (particularly sleep fragmentation), daytime energy (especially fatigue and excessive daytime sleepiness), and nighttime oxygen saturation are priority targets for digital measure development. Third, smartwatches, sleep mats, and smart rings are strongly preferred as modalities for sDHTs that can be integrated into future RPM solutions. Fourth, current digital monitoring practices should be enhanced by focusing on expanding metrics beyond AHI, improving measurement accuracy, and ensuring that digital measures are meaningful, understandable, and actionable for end users. Finally, patients lack the ability to determine from RPM output whether they need to seek follow-up care and, if so, what type of care is appropriate.

### Comparison With Previous Work

Our principal findings highlight that improving subjective sleep quality, increasing physical activity, and enhancing daytime energy levels are key meaningful aspects of health for patients with OSA. Each can (potentially) be assessed using sDHT-derived metrics, though these technologies vary widely in maturity and clinical readiness.

Sleep was a consistently prioritized health aspect, essential to daily functioning and overall well-being. Participants expressed skepticism about the accuracy and clinical utility of sleep data from personal devices, echoing World Sleep Society recommendations that, while sDHTs hold potential for monitoring sleep patterns, their proprietary algorithms often lack validation in sleep disorders [37]. Recent studies in OSA populations show promising results for tracking metrics like total sleep time and sleep efficiency, with some devices showing moderate-to-strong agreement with polysomnography; however, broader validation is still needed to ensure accuracy and clinical relevance [38-42].

Physical activity emerged as another key health aspect, with participants identifying it as a health priority and an area they wished to improve. Physical activity is both a meaningful health goal and a key factor in OSA management, as increasing activity can help reduce OSA severity while effective treatment may, in turn, support higher activity levels. Structured exercise interventions can reduce AHI, improve oxygenation, and alleviate daytime sleepiness [43-45]. However, evidence for physical activity levels as an outcome of CPAP therapy is mixed, with studies reporting, despite improved symptoms, modest or no changes in physical activity levels [46-48]. sDHTs could help monitor physical activity as a potential biomarker of functional gains and support behavior change in OSA care.

Daytime energy levels emerged as a top-rated health aspect, with participants identifying it as a key priority, an important health goal, and the most valued quality-of-life factor to restore. This reflects both excessive daytime sleepiness and fatigue, which present heterogeneously across patients and persist in some even after optimal therapy [49-51]. While these symptoms are typically assessed using PROMs, such as the Epworth Sleepiness Scale and Fatigue Severity Scale, their use is limited by respondent burden, recall bias, and low temporal resolution [51]. sDHTs offer a promising, noninvasive alternative for continuous monitoring of these symptoms. Metrics, such as heart rate variability and physical activity, are actively being explored, yet validated digital measures for routine monitoring are lacking—making this a clear priority for innovation [51,52].

### Implications for Practice and Policy

#### For Developers of sDHTs and RPM Solutions

Developing new digital measures and RPM solutions is complex and resource-intensive, often requiring significant time before their impact reaches clinical practice. In the short term, the rapid rise of consumer wearables creates an opportunity to validate and optimize digital measures—such as sleep and physical activity—for specific populations like people with OSA. This should be prioritized as a practical step toward innovation. Beyond creating new measures, improving existing RPM tools is equally urgent. Enhancing reporting platforms with clear explanations, contextualized feedback, and actionable suggestions could better support patients in managing their condition. Interfaces that let users toggle between simplified and detailed views would help accommodate diverse preferences and levels of health literacy.

#### For Policymakers and Payers

On a broader scale, policymakers and payers should consider setting standards to ensure RPM systems deliver accurate, patient-centric, harmonized, and actionable data presentations. A key requirement is that digital measures embedded in RPM solutions are validated for accuracy, reliability, usability, and clinical relevance in target populations, such as patients with OSA [22,23]. Establishing such validation criteria will help ensure that these tools provide meaningful insights and support clinical decision-making. These standards could also guide reimbursement decisions and accelerate the adoption of RPM solutions that empower patients, improve treatment outcomes, and reduce health care burden.

#### For Clinical Practice

Our findings underscore several opportunities to strengthen clinical care. First, follow-up care for patients with OSA is often limited and fragmented, highlighting the need for better alignment between home care providers and clinicians and a clearer definition of follow-up pathways. Telemedicine offers a promising, cost-effective avenue to facilitate structured and timely follow-up. Second, patients frequently report difficulties in interpreting RPM data; clinicians and home care providers should therefore provide guidance to ensure patients understand the outputs from their CPAP. Finally, as patients increasingly adopt self-monitoring solutions outside formal care pathways, clinicians should be supported in evaluating the reliability of these tools and integrating relevant patient-generated data into care when appropriate.

### Limitations and Strengths

Our study has several limitations. First, the sample was skewed toward more highly educated individuals and included a higher proportion of women, likely reflecting the greater prevalence of comorbid insomnia in female patients with OSA [53-55]. Second, the survey was not pretested, which might impact its validity. Third, the number of participating health care professionals was limited to 3 (1 somnologist, 1 somnologist/pulmonologist, and 1 general practitioner), which means their perspectives may not fully capture the diversity of clinical views and could reflect individual experiences. However, several of their insights were echoed by patients, supporting their relevance to the themes identified. Furthermore, the surveyed cohort focused on individuals with OSA who report persistent sleep problems despite treatment. While this subgroup may not fully represent the broader OSA population, it highlights a group with substantial unmet needs and provides valuable insights into priorities for digital health innovations and RPM solutions. Another strength is that our patient-centric approach aligns with value-based health care principles and was guided by the digital measures that matter framework to identify outcomes that matter most to individuals with OSA. The combination of quantitative survey data and qualitative interviews, including input from patient representatives, ensured the perspectives captured reflect a broad OSA population.

### Conclusion

Rather than relying solely on clinical end points such as AHI, our findings suggest that outcomes such as physical activity, restorative sleep, and daily functioning are central to patients’ lived experiences—and are therefore critical targets for sDHTs and RPM metric innovation. Developing and validating new digital measures that capture these experiences will require time, interdisciplinary collaboration, and ongoing involvement of patients to ensure relevance and usability. In the meantime, existing RPM systems can be strengthened by improving transparency, accessibility, and contextual interpretation of currently collected data, making these platforms more meaningful and actionable for patients. The broad patient priorities identified in this study can serve as an excellent starting point for defining patient-centric digital measures, allowing for comprehensive disease management.

## Data Availability

The datasets generated and analyzed during this study are publicly available in the Harvard Dataverse repository under the title “Replication Data for: Toward Patient-Centric Digital Health Solutions for Obstructive Sleep Apnea Monitoring: Perspectives from Dutch Patients and Healthcare Professionals – a mixed-method study” [36].

## Appendix

Multimedia Appendix 1 Overview of themes covered in mixed methods components, characteristics of interviewed cohort (n=11), survey (original in Dutch, for this manuscript translated to English), and interview guide (original in Dutch, for this manuscript translated to English).

Multimedia Appendix 2 Mixed Methods Reporting in Rehabilitation and Health Sciences checklist.

Multimedia Appendix 3 Survey response flow diagram. The final sample was defined as respondents who completed demographic questions (Q1-8) and at least one study question (Q9-18).

## Abbreviations

AHI: Apnea-Hypopnea Index
CAQDAS: computer-assisted qualitative data analysis software
CPAP: continuous positive airway pressure
DiMe: Digital Medicine Society
MAD: mandibular advancement device
OSA: obstructive sleep apnea
PROM: patient-reported outcome measure
RPM: remote patient monitoring
sDHT: sensor-based digital health technology
TNO: Netherlands Organization for Applied Scientific Research
