# Supplementary material for: Toward Patient-Centric Digital Monitoring of Obstructive Sleep Apnea: Mixed Methods Study
Source: J Med Internet Res. 2026 Jan 8;28:e82460. doi: 10.2196/82460 (PMC12828318; doi:10.2196/82460)
Supplement: Multimedia Appendix 1 [file jmir_v28i1e82460_app1.docx]

**Table S1. Characteristics of interviewed cohort (n=11)**

| ID | Sex | Stakeholder group |
| --- | --- | --- |
| B1 | Female | OSA patient |
| B2 | Male | OSA patient |
| B3 | Female | OSA patient |
| B4 | Male | OSA patient |
| B5 | Male | OSA patient |
| B6 | Male | OSA patient |
| B7 | Male | Somnologist |
| B8 | Male | General practitioner and patient |
| B9 | Female | Pulmonologist/somnologist |
| B10 | Female | Patient advocate |
| B11 | Male | Patient advocate |

OSA: obstructive sleep apnea.

**Table S2.** **Overview of themes covered in mixed methods components.**

| **Theme** | **Survey** | **Interview** |
| --- | --- | --- |
| Meaningful aspects of health | Yes | No |
| OSA^a^ follow-up care in NL | Not explicitly queried—but some responses to the open-ended questions provided further information. | Yes |
| Attitudes toward RPM^b^ and currently used sDHTs^c^ | Focus on sDHTs | Focus on RPM |
| OSA-related health metrics priorities | Yes | Yes |
| Future of sDHT-based OSA RPM | Yes | Yes |

^a^OSA: obstructive sleep apnea.

^b^RPM: remote patient monitoring.

^c^sDHT: sensor-based digital health technology.

**Supplementary file 1. Survey (original in Dutch, for this manuscript translated to English)**

Questions 1 to 4 are about general background information.

1. What is your gender?
 Female
 Male
 Other
 Prefer not to say

2. What is your age?
______________________________

3. What is your highest level of education?
 Primary & lower secondary education (e.g., elementary school, pre-vocational education)
 Higher secondary education (e.g., high school, vocational training)
 Higher education (e.g., college or university degree)

4. It is known that irregular work and night shifts affect sleep. Do you work at least one day per week? If yes, which of the following best describes your current work schedule?
 Regular day shifts (mostly during the day)
 Night shifts (mostly at night)
 Shift work including night shifts
 Shift work excluding night shifts
 I do not work or work less than one day per week

Section 5

Questions 5 through 8 are about your sleep problems.

5. Has a healthcare provider diagnosed you with any of the following sleep problems?
(Multiple answers possible)
• Insomnia
• Sleep-related breathing disorders, such as sleep apnea
• Excessive daytime sleepiness (Hypersomnia)
• Circadian rhythm sleep disorders (e.g., delayed sleep phase disorder, shift work disorder)
• Parasomnias (e.g., sleepwalking, nightmares, bedwetting)
• Sleep-related movement disorders (e.g., restless legs syndrome)
• Another diagnosis, namely: ______________________________
• No formal diagnosis has been made by a healthcare provider

6. Do you have any other illnesses or health risks?
(Multiple answers possible)
• No
• Type 2 diabetes
• (Risk of) cardiovascular disease
• Depression
• Cancer
• Obesity
• Other, namely: ______________________________

7. Which healthcare provider(s) have you visited in the past year for your sleep problem?
(Multiple answers possible)
• General practitioner
• Medical specialist (e.g., pulmonologist, cardiologist, neurologist)
• Occupational physician
• Psychologist
• Sleep therapist
• Sleep center
• Other, namely: ______________________________
• I have not visited a healthcare provider for my sleep problem

8. What type(s) of treatment are you currently receiving for your sleep problem?
(Multiple answers possible)
• I received advice on lifestyle changes to improve sleep (e.g., exercise, diet, daily routines)
• I receive support for sleep improvement through work adjustments
• I receive psychotherapy focused on sleep (e.g., cognitive behavioral therapy)
• I use a device to support breathing (e.g., CPAP or mandibular advancement device)
• I use sleep medication
• Other, namely: ______________________________
• I am not currently receiving any treatment

Section 6

9. Can you list one or more activities you used to be able to do before your sleep problems started that you would like to do again?
______________________________

10. What are the three most bothersome aspects of your sleep problem?
Rank your top three by clicking on the options and adjusting the order if necessary:
• Difficulty concentrating
• Falling asleep during (long) car rides
• unable to think clearly
• Impact on relationships with family and friends
• Mood swings
• Too tired for hobbies or sports
• Getting through the night
• Sleep (or lack of it) dominates my life
• Worrying about the impact of sleep problems on overall health
• Other

10a. If you selected "Other" in the previous question, what did you mean?
______________________________

11. Can you list one or more things you would like to improve about your health and/or sleep problem?
______________________________

12. Which two aspects of health are most important to you?
Select your top two by clicking and reordering if necessary:
• Physical fitness and ease of movement
• Mental and emotional wellbeing
• Quality of sleep and rest
• Eating habits
• Management of chronic conditions (if applicable)
• Other, namely: __________________________

Section 7

13. Which three aspects of your sleep problem would you like to better understand?
Rank your top three:
• Sleep characteristics: time to fall asleep, total sleep duration, sleep quality (deep/REM sleep), waking up during the night
• Better understanding of how my sleep problems develop over time
• Daytime energy/fatigue
• Heart rate, breathing, oxygen saturation
• Muscle tension
• Sleep environment (light, temperature, air quality)
• Daily physical activity
• Eating and drinking habits
• Overall health to better understand factors influencing sleep
• Other

13a. If you selected "Other" in the previous question, what would you like to better understand?
______________________________

14. Do you believe digital technology can help improve your (sleep) health?
 Yes
 No

14a. If “No” was selected: Why do you believe digital technology cannot help improve your (sleep) health?
______________________________

Section 8

15. Are you currently using any technology to monitor your health and/or sleep?
(Multiple answers possible)
• Scale
• Blood pressure monitor
• Heart rate monitor (e.g., smartwatch or chest strap)
• Sleep tracking with smartwatch or ring (e.g., Apple Watch, Fitbit, Oura)
• Sleep mat or strip in the bed
• Oxygen saturation monitor
• CPAP device
• Glucose meter
• Health app (e.g., for diet)
• Other, namely: ______________________________
• No

16. What could be improved about the measurements you currently use?
______________________________

17. Who should be able to access your recorded data?
 Only myself
 Only my healthcare provider
 Myself and, in some (user-defined) cases, my healthcare provider
 Both myself and my healthcare provider

18. Below are images of what the technology TNO is developing might look like. Please rank them by dragging, placing the most appealing option at the top:
• Sleep mat


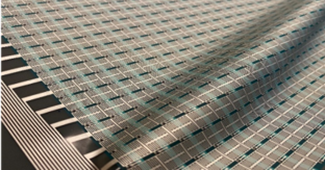

• Sleep strip on the mattress


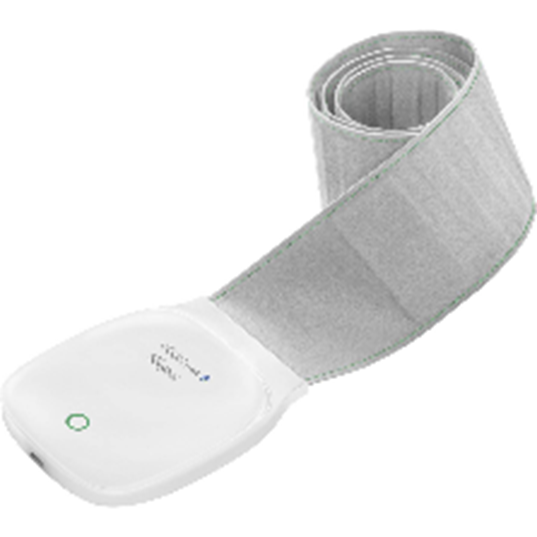

• Ring


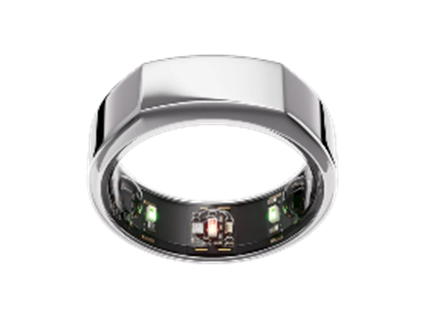

• Clothing


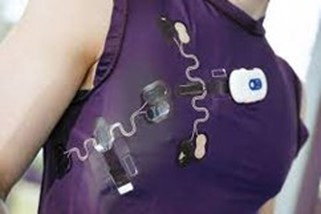

• Watch


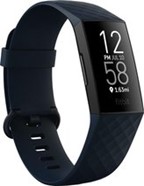

• Patch


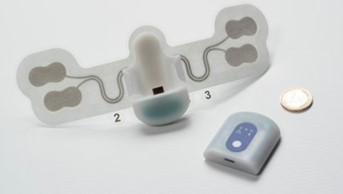

• Headband


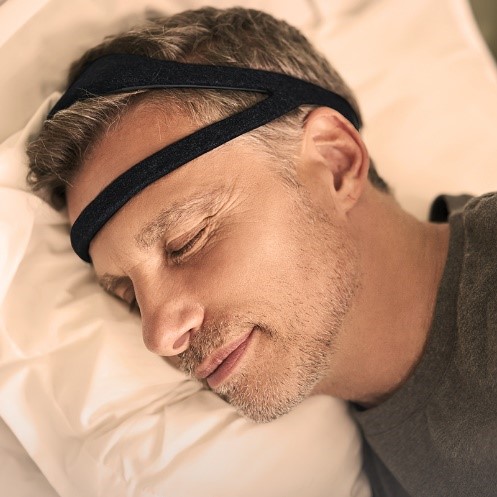


**Supplementary file 2. Interview guide (original in Dutch, for this manuscript translated to English)**

**Demographic information**

1. What is your name and age?
2. What is your occupation?
3. What is your education level?
4. Who is your current physician for your sleep apnea management or from whom did you receive patient care for your sleep apnea?

**Remote patient monitoring (remote patient care)***Refers to the practice of monitoring and treating sleep apnea using remote technologies and methods, rather than through traditional in-person visits to healthcare facilities. This approach typically involves the use of remote patient monitoring devices, telemedicine consultations, and digital health platforms to assess and manage sleep apnea symptoms, track treatment progress, and provide ongoing care to patients from a distance.*

1. What type(s) of remote patient monitoring tool(s) are used during sleep apnea management?

- Reasons for using/not using.
- Concerns regarding RPM tools
- Overall opinion about RPM tools in healthcare

**Follow-up care during remote patient care**

*With follow-up care we mean the ongoing care and support that patients remotely receive after being diagnosed, so e.g. from home or another location outside the traditional healthcare setting.*

1. What does the follow-up care for sleep apnea look like?

- With whom contact? (current physician for sleep apnea management)
- Frequency of information exchange (communication) (per week, month, year)
- Frequency of receiving/sending reports (per week, month, year)
- Satisfaction/unsatisfaction

1. If there is no follow-up care at all, how does communication and reporting take place?

- Reasons for absence of follow-up care
- Need for follow-up care
- Reasons for absence of communication and reporting 🡪 If so, continue with:

1. What recommendations do you have to improve the current follow-up care? Or: what does the ideal follow-up care look like for you?

**Needs and opportunities regarding current communication & reporting mechanisms in RPM**

1. What are your needs concerning the current communication and reporting mechanisms during remote patient care?

- Needs to ***understand*** the information better.
- Needs to ***access*** the information better.
- Needs concerning the ***type of information*** that is being communicated/reported.
- Needs concerning ***how information*** is communicated/reported.

1. What suggestions or recommendations do you have to improve the current communication and reporting mechanisms used during remote patient care?

**Needs concerning communication & reporting mechanisms for future RPM technologies**

1. If you could design the perfect remote patient monitoring (RPM) device for sleep apnea, what would the ideal communication and reporting mechanism look like for you?
