# Supplementary figures and images for: Toward Patient-Centric Digital Monitoring of Obstructive Sleep Apnea: Mixed Methods Study
Source: J Med Internet Res. 2026 Jan 8;28:e82460. doi: 10.2196/82460 (PMC12828318; doi:10.2196/82460)

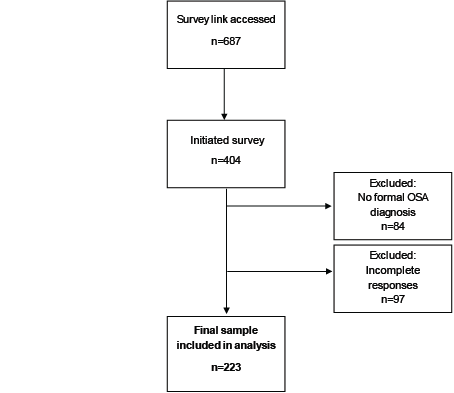

Supplement: Multimedia Appendix 3 [file jmir_v28i1e82460_app3.png]
